# Functioning of unidirectional ventilation in flying hawkmoths evaluated by pressure and oxygen measurements and X-ray video and tomography

**DOI:** 10.1242/jeb.245949

**Published:** 2024-09-17

**Authors:** Lutz Thilo Wasserthal, Peter Cloetens

**Affiliations:** ^1^ Department of Biology, University of Erlangen-Nuremberg, Staudtstr. 5, D-91056 Erlangen, Germany; ^2^ European Synchrotron Radiation Facility, 71 Avenue des Martyrs, F-38043 Grenoble, France

**Keywords:** Respiration, Air sacs, *Acherontia*, *Manduca*, *Xanthopan*

## Abstract

Flying sphingids generate unidirectional ventilation with an inflow through the anterior thoracic spiracles and an outflow through the posterior thoracic spiracles. This phenomenon was documented by the CO_2_ emission and tracheal air pressure in split-chamber experiments in preceding studies. In the present study, we evaluated the function of the air pump mechanism by measuring the tracheal pressure and *P*_O_2__ in the air sacs and monitoring the wing beat using photocells. Microelectrodes recorded the abdomen flexing muscles and abdominal transverse muscle septum. The crucial structure was the vertical mesophragma, with longitudinal flight muscles attached anteriorly and large fused metathoracic air sacs posteriorly, continuous to the first abdominal segment. Longitudinal flight muscles and abdomen lifting muscles contracted synchronously, producing positive pressure pulses within the mesothoracic air sacs. In the scutellar air sacs, the *P*_O_2__ with starting full flight was elevated to 18–20 kPa, with a pressure increase of 35–50 Pa. In contrast, in the metathoracic air sacs, the O_2_ concentration during flight could rise to 10 kPa, then decline to 5±1 kPa. The metathoracic air sacs provided compliance for ventilation by the flight muscles. The initial rise and subsequent decrease of the *P*_O_2__ in these posterior metathoracic air sacs indicated the unidirectional flow path of the air used. Serial X-ray frames of flying *Acherontia atropos* visualised the cyclic phragma movement and volume changes in the metathoracic air sacs. The results showed that the contracting dorsolongitudinal flight muscles expanded the metathoracic air sacs, acting as a suction pump.

## Teaser

**Summary:** Longitudinal flight muscles are attached to the movable vertical mesophragm, providing sucking force in the air sacs behind for unidirectional ventilation.

## INTRODUCTION

Sphingid moths are known for their powerful and enduring flights. They hover before the blossoms during flower visits, sucking nectar through their long proboscis. Their persistent flight performance is undeniable in migrating species, such as *Agrius convolvuli*, *Acherontia atropos*, *Hippotion celerio* and *Macroglossum stellatarum*, known to migrate to Europe from the Mediterranean and Africa (Reinhardt and Harz, 1996). *Acherontia atropos* sphingids are specialised to invade beehives to suck honey from the bee combs. Other species, such as *Xanthopan morganii* and *A. convolvuli*, developed extremely long proboscises capable of exploiting nectar from deep corolla tubes.

The ability to hover on the spot attracted scientific interest, focusing on the anatomy and function of the indirect flight muscles (Eaton, 1988; Pringle, 1975; Gau et al., 2019), wing movements (kinematics and aerodynamics), and their influence on the airflow around the wings (Combes and Daniel, 2003; Demoll, 1918; Heinig, 1982; Usherwood and Ellington, 2002; Van den Berg and Ellington, 1997; Willmott et al., 1997). The moths stabilise their flight by flexing their body axis owing to a joint between the thorax and abdomen (Dyhr et al., 2013). Among the Lepidoptera, in hawkmoths with a relatively high wingbeat frequency of 27–73 Hz (Weis-Fogh, 1964b) and small amplitude, abdominal movements are inconspicuous to the naked eye. It has been discussed whether the wingbeat-coupled abdominal movements were only artefacts owing to tethering. This problem has been addressed in a study about the role of flexing the abdomen for flight stabilisation under varying optomotor reactions (Dyhr et al., 2013).

Unidirectional ventilation during flight has been investigated in sphinx moths. In a previous paper (Wasserthal, 2001), CO_2_ emission was measured in a flow-through chamber using ultrared radiation analysis spectrometry (URAS). The flow of CO_2_-depleted air, regulated for a calibrated time volume, was used to resolve single ventilatory pulses. The intratracheal flow path was evaluated using a two-chamber arrangement. The anterior spiracles (SP I) were equipped with tubes glued around the outer margin of the intact spiracles. The tube from each spiracle could be connected independently to a pressure sensor, or both tubes could be connected to a smaller compartment of the split chamber, and their outputs could be combined for either pressure or CO_2_ emission measurements. The moths with metathoracic spiracles (SP II) were in the main compartment (Wasserthal, 2001). CO_2_ outflow was only recorded through SP II. Negative pressure measured at SP I indicated suction for inflow.

We hypothesised that special anatomic–functional coupling of the contracting flight muscles to internal tracheal structures must be present to induce directed flow. The anatomical description of the tracheal system of the sphingid *Manduca sexta* is incomplete (Eaton, 1988). That author described SP II as dysfunctional for being enclosed in the intersegmental fold, and was unaware that these spiracles opened during the upstroke of the wings. Based on a preceding paper (Wasserthal, 2001), it was hypothesised that these spiracles play a crucial role in directing ventilatory flow.

**Fig. 1.**
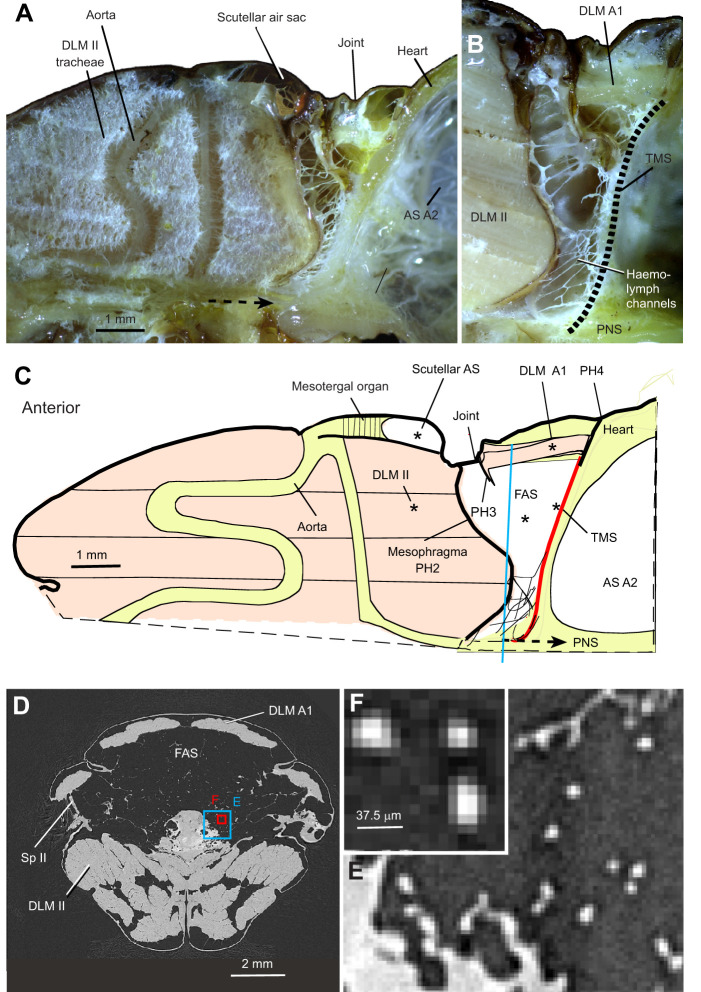
**Anatomy of the metathoracic–abdominal region containing large air sacs.** (A,B) Thorax and anterior abdomen of *Xanthopan morganii* (male, 1/04) dissected along the parasagittal plane, showing the dorsal internal right half with the fused thoracoabdominal air sacs (FAS). (B) Close-up immediately at the right of the midline, showing the dorsolongitudinal flight muscles (DLM II) and supposed haemolymph channels traversing the FAS. Dotted line: position of the transverse muscle septum (TMS) containing the dorsoventral muscles of the first abdominal segment. (C) Schematic longitudinal view of the dorsal thorax and anterior abdomen of *Acherontia atropos*. Asterisks point at the positions of the sensors and microelectrodes, respectively. DLM A1, dorsolongitudinal muscles of the first abdominal segment; PNS, perineural sinus; PH3, cuticular phragma among the third thoracic and first abdominal segment; PH4, cuticular phragma between the first and second abdominal segment. (D) X-ray tomographic cross-section through the FAS (blue line in C). (E,F) Overview with boxed regions showing cross-sections of haemolymph channels. The X-ray slice of these tubes shows the highest density in the centre of the channels, containing tissue and possibly fluid.

The present paper provides insight into the working system that generates unidirectional ventilation. We evaluated the mechanism of the pressure and suction pump producing airflow during flight. Intratracheal ventilation was analysed by applying oxygen optodes and pressure sensors. The fluctuations in tracheal pressure and the partial pressure of oxygen (*P*_O_2__) were evaluated for shivering and steady flight, measured in the (meso-)scutellar air sacs or the thoracoabdominal air sacs within the waist. Simultaneous electrophysiological recording of flight muscles and abdominal flexing muscles was performed synchronously with pressure measurements in the scutellar air sacs or with the muscles of the abdominal phragma (Fig. 1, asterisks indicate sensor positions). Serial radiographies taken during flight visualised movement of air sacs and protraction of the mesophragm during downstroke without any of the physiological recordings. The opening and closing of the SP II during the wing beat cycle was visualised using slow-motion videography.

**List of abbreviations**

AS A2: air sac of abdominal segment 2
DLM II: dorso-longitudinal muscle of thorax segment II thoracic wing depressor
DLM IIT: dorso-longitudinal muscle tracheae
DLM A1: dorso-longitudinal muscle of abdominal segment 1 (abdomen lifting muscle)
DVM II: dorso-ventral muscle of the mesothorax (wing elevator muscle)
FAS: fused thoracoabdominal air sac
PH2: mesophragma
PH3: phragma in the third thorax segment
PH4: phragma in the anterior abdomen
PNS: perineural sinus
SA: scutellar air sac
SP I: spiracle I of the thorax
SP II: spiracle II of the thorax
TMS: transverse muscle septum
MS1: muscle M 1 of transverse muscle septum
MS2: muscle M 2 of transverse muscle septum

## MATERIALS AND METHODS

### Animals and handling procedures

We used large species and strong flyers for physiological and anatomical studies. As commercially obtainable lab stocks often suffer reduced vitality owing to inbreeding, we preferred offspring from field-captured females. The adult moths were also captured at potted nectar plants in the garden, attracted by wild or artificial lily-shaped flowers with a 12–18% honey–water solution. Only long-lived and persistent flyers or their direct offspring were used in the experiments. The hawkmoths were kept inside large cube-formed flight cages of 2×2×2 m within an air-conditioned greenhouse of the University of Erlangen-Nuremberg. They were allowed to feed from artificial flowers with an 18% honey solution. Host plants were provided for egg laying. The caterpillars were reared in plastic boxes of 5 l volume with cut twigs. Larvae were fed on their natural host plants.

The following hawkmoth species were held as stocks for several years: *Agrius convolvuli* (Linnaeus 1758) was bred on *Convolvulus arvensis* and *Ipomoea* (Convolvulaceae); *Acherontia atropos* (Linnaeus 1758) was fed on *Ligustrum ovalifolium* (Oleaceae); *Manduca sexta* (Linnaeus 1763) imported from la Selva, Costa Rica, was fed on *Nicotiana* (Solanaceae); and *Hippotion celerio* (Linnaeus 1758) and *Macroglossum stellatarum* (Linnaeus 1758) were fed on *Galium verum* (Rubiaceae). Finally, *Xanthopan morganii* (Walker 1856) was reared from eggs collected in Madagascar and raised on greenhouse trees *Annona muricata* (Annonaceae)*.*

For experiments, 5- to 10-day-old healthy moths (body mass range 1.4–3.0 g) were selected. For all combinations of sensors, at least three specimens were measured for 3–5 days.

Before preparation, the moths were anesthetised for a few minutes in CO_2_ gas. The body scales were locally removed before puncturing the integument and fixation of the adapter cone as the tether (see below). All of the following preparatory steps were conducted gently without narcosis, allowing recovery periods of several hours.

After suspension, the moths were offered foot contact with a Styrofoam ball of 200 mm and 0.11 g in a round vessel below for rest. They lost foot contact when starting the flight, which was elicited by dimming the light. A nocturnal activity rhythm was used. The moths remained suspended for the entire experimental period over 3–5 days, with continuous registration of the sensor parameters. In addition, spontaneous flight activity was included.

The moths repeatedly flew for several minutes without further encouragement, such as using a fan or moving optical patterns. During tethering, the moths were fed *ad libitum* with a 12–18% honey–water solution before the beginning, between and after the sessions, which lasted a few minutes to several hours. At the end of the experiments, the moths were still healthy and could be separated from the mounting rod or even released.

Data were acquired using an eight-channel PowerLab/8SP-8-I (ADInstruments, Oxford). Calculations were performed using Chart 3.63 software on Power Macintosh computers.

### Anatomy

The anatomy of the hawkmoths was studied by dissecting large individuals of *X. morganii*, *A. convolvuli*, *A. atropos* and *M. sexta* prefixed by fumigating the CO_2_ narcotised moths with 37% formaldehyde vapour.

### Photocell records of moving wings and abdomen

The experiments were performed in a Faraday cage at 20±1°C. The wingbeat was recorded by projecting the shadow of the moving wings onto a silicon photocell (Conrad Hirschau, Germany 55×20 mm or Telefunken BPY 10×3 mm) installed below, sideward of the moths. Illumination was made using a fibre optic lamp (Schott, Mainz, Germany). For high temporal resolution, the sampling rate was increased to 40 kHz. In contrast, 400 Hz was used during long-term measurements. The electronic processing of the photocell voltage signal showed a delay of 2.5–3 ms relative to the pressure pulse, which was accounted for in the analysis of the recordings. The abdominal lifting and lowering movements were recorded using a self-adhesive reflex tape (Scotchlite S.O.L.A.S., 10×3 mm, 3M Science Applied to Life, Neuss, Germany) applied to the abdominal tip. The light for the laterally arranged photocell was interrupted or reflected by the vertically upward and downward moving caudal tip. Fibre optic illumination was similar as described before. The recording was made with a custom-made electronic setup.

### Tracheal pressure measurements

**Fig. 2.**
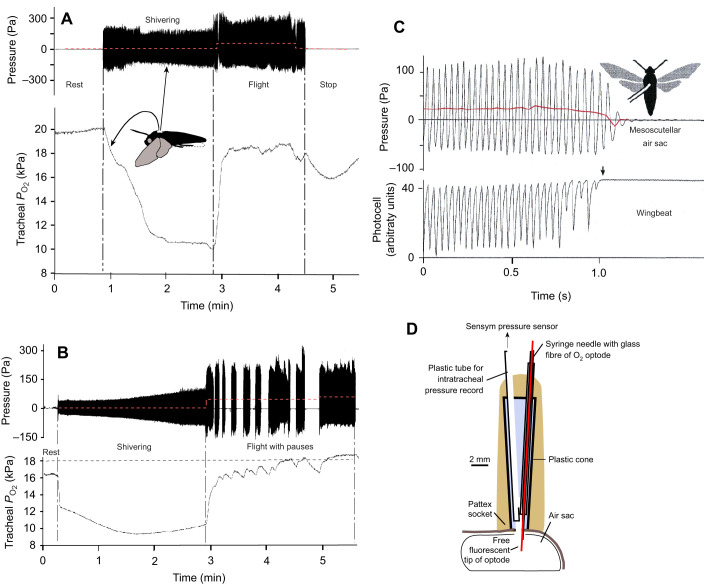
**In the scutellar air sacs, tracheal *P*_O_2_ _declines during shivering, rising to nearly ambient *P*_O_2_ _under rising pressure during sustained flight.** (A,B) Simultaneous measurements in the scutellar air sacs of (A) *Manduca sexta* (female, 6/04, *N*=6, *n*=125) and (B) *Agrius convolvuli* (male, 2/03, *N*=5, *n*=72). (C) Upper trace shows pressure pulses in the scutellar air sacs correlate to flight muscle activity. The lower trace shows the movements of the wings, determined by photocell recording, which can be influenced by manoeuvring attempts, resulting in one-sided weak wingbeats (*M. sexta* female, 2/00, *N*=6). In A–C, dashed red lines mean tracheal pressure. (D) Graph of the adapter cone with the inserted tube for connection to the pressure sensor and optode for O_2_ sensing (modified from Wasserthal, 2014).

Pressure sensors Sensym SCXL 004 DN (Hawker and Siddeley, London, now Honeywell) were used. The pressure sensors were precalibrated to absolute values of 0–70°C and 0–100% humidity. Pressure values were controlled in the experimental setup using a bifurcated tube arrangement, with the pressure sensor at one end and the other connected to a calibration pressure sensor (Manocal, model HQS-1 0.6% in the range of 0–10 Mbar, S/N HQS −19897, Dresser Instrument Division, Newtown, CT, USA). A hole was cut for sensor preparation by puncturing the cuticle and the underlying air sacs using an ophthalmic knife and scissors. First, a plastic adapter cone (tip of an Eppendorf pipette with an inner diameter of 1.6 mm and outer diameter of 2.3 mm) was fixed as a mounting rod with Pattex glue (Henkel, Düsseldorf, Germany). The cone served as a tether. Microcatheter PVC tubes (Reichelt Chemietechnik, Heidelberg, Germany) (1.2 mm external and 0.8 mm internal diameters) were inserted into the cone. The cone had enough space to insert another sensor, such as an O_2_ optode. The cone was tightly sealed on the cuticle using Fixogum rubber cement (Marabu, Tamm, Germany). The tubes allowed the moth to be connected to the pressure sensors (Fig. 2D; Fig. S1) (Wasserthal, 2015, fig. 11).

The tubes were glued to the adapter cone to prevent positional changes of the tube relative to the air sac, which could cause artificial pressure pulses (graphics in Wasserthal, 2014) (Fig. 2D). The delay between mechanically applied pulses and electronic pressure registration was approximately 1 ms for air pressure. The tube connecting to the pressure sensor varied from 48 to 81 mm in length. These differences in tube length had no measurable effect (delay or dampening of signal) when changing from shorter to longer tubes. Optimal flight temperature was attained in the experiments after the transition from shivering to full flight (Kammer, 1968; Komai, 1998).

### *P*_O_2__ measurements

The tracheal *P*_O_2__ measurement was performed using the micro fibre optic oxygen transmitter Microx TX3 AOT (PreSens Precision Sensing GmbH, 93053 Regensburg, Germany). The tapered tip (diameter 50 μm) of this fibre was oriented directly above the perforation or inside the air sacs. The fibres were arranged beside the air pressure tube within the adapter cone described above. The measurements were run under controlled ambient temperature, at 20±1°C. The sampling rate of the optode was 1 Hz. The response time was 40 ms, and the time constant (interval from 17.4 to 20 kPa) in the experimental setup was 1.5 s. Calibrations in the O_2_-free and 100% O_2_ treatments were repeated before and after each experiment. The stability of the optodes allowed continuous use over several weeks without significant reduction in sensitivity, with only a slow, gradual loss in response time when used in air. For further details, see Wasserthal (2014, fig. 1).

### Electrophysiological recordings

Recordings of the indirect flight muscles were performed during the tethered flight. The action potentials were measured using paired V2A-steel microelectrodes (20 µm diameter) inserted into the wing depressor DLM II, the abdomen flexing muscle, the DLM A1 or the transverse muscle septum TMS with two muscles, TMS1 and TMS2 (asterisks in Fig. 1C).

### Data acquisition

Data were continuously recorded on an Apple Powermac or PowerBook using a custom-made amplifier and a PowerLab S-channel Interface with software (Chart 5.54: CH Sciences, Milford, MA, USA).

### Slow-motion videography

The opening and closing of the metathoracic spiracle was visualised using a Sony Alpha 9 camera (ILCE-9 2017, firmware updated September 2019 to version 5.01). The moths were partially descaled on the thorax to better visualise the spiracle opening. The frame rate could be modified using the slow-motion option, applying the fixed frame rates of 120, 60, 30 and 15 frames s^−1^. The appropriate frame rate was a value slightly lower than the wingbeat frequency, or the integer fraction thereof. When the animal varied its wingbeat frequency to a coincidentally suitable frequency, a uniform slow movement of the spiracle was visible on the camera screen, using the stroboscopic effect. This was successful when applying 30 frames s^−1^, and the even movement indicated that the moth transiently elevated the wingbeat frequency slightly above 30 wingbeats s^−1^. Under the stroboscopic interference conditions, every visualised cyclic spiracle movement was based on 123 frames, every frame caught slightly later with respect to the wingbeat cycle. This required longer footage to reach a satisfactory result.

The wingbeat frequency was deduced from the frame rate of 30 frames s^−1^. The stroboscopic slow-motion effect took place with 124 wingbeats within 4.1 s: one wingbeat more than the 4.1×30 frames in that timespan. Hence the actual wingbeat frequency was 124/4.1 s=30.24 Hz. The video does not represent the spiracle opening and closing movement during single wingbeat cycles. It shows photos made during consecutive wingbeats, every frame photographed slightly later than in the preceding spiracle opening and closing cycle.

The wingbeat frequency in *A. atropos* normally varied between 20 and 27 Hz (*N*=2). The wingbeat correlated with abdomen lifting was documented in *A. atropos* (*N*=3) using the slow-motion option with 25 frames s^−1^.

In *M. sexta*, *M. stellatarum*, *H. celerio* and *A. Atropos*, abdomen lifting during wingbeat was documented by a 16 mm Arriflex high-speed Cine camera (München, Germany) under stroboscopic light (Drelloscope 1018 So 16 II, Mönchengladbach, Germany) with a frame rate of 100–150 Hz.

### Recording of X-ray micro-CTs

X-ray investigations of hawkmoths were conducted at the European Synchrotron Radiation Facility (ESRF) Grenoble, France, at beamline ID19 in February 2005. Synchrotron X-ray phase-contrast imaging was used. The technique was developed by ESRF Grenoble and by Argonne National Laboratory, USA (Westneat et al., 2003). For the tomography, *A. atropos* moths (*N*=3), after being anesthetised in CO_2_ gas, were put into a vessel with 40% formaldehyde for 2 h for fixation. Monochromatic synchrotron radiation (15 keV photon energy from a double-crystal silicon monochromator) was used. The indirect X-ray detector consisted of a thin gadolinium oxysulfide (Gd_2_O_2_S) scintillator screen, with a lens coupled to an ESRF-made CCD camera (FReLoN with ATMEL chip 7899M) (Labiche et al., 2007). The pixel size of the detector was 7.46 μm, and the projection size was 2048×2048 pixels, resulting in a maximum field of view of 15 mm. The exposure time was 0.3 s per radiograph, and 1500 radiographs were recorded during a turn of 180 deg. Recorded at distances of 50 mm, the radiographs contained both absorption and phase information. Tomography reconstruction was performed using the filtered back-projection algorithm as implemented in the ESRF software PyHST. A total of 4×256 slices (1024) were further analysed per specimen with a final spatial resolution of the images of 12 μm. The datasets, derived from a 2 cm length for the head and thorax, were analysed with NIH ImageJ (Schneider et al., 2012) on a G5 Macintosh computer. The tomographic data were visualised by segmenting the data using the segmentation tool of Amira (Visual Concepts GmbH, Berlin, Germany), which resliced the tomographic stacks along the longitudinal axis of the insects.

### Serial X-ray frames taken during flight to document the movement of the mesophragm

In 2005, real-time X-ray high speed videography had not yet been developed (Socha et al., 2007). Serial radiographs taken during flight were used to create a slow-motion view of the metathoracic–abdominal air sacs and mesophragm. *Acherontia atropos* moths (*N*=3) were suspended by a tether and given foot contact with a Styrofoam ball. Removal of the ball elicited flight.

The movement of the mesophragm, expanding the air sacs behind, was visualised based on a series of 300 X-ray frames, exposure 5 ms with 1 frame s^−1^ during 5 min of full flight to minimise the radiation impact. Monochromatic radiation with an energy of 15 keV and a sample-to-detector distance of 200 mm was used. The same FReLoN based X-ray detector as for the X-ray micro-CTs was used (see above). However, the radiographs were binned twice, resulting in a frame size of 1024×1024 pixels (pixel size 15 μm). All acquisition parameters were carefully selected to minimise radiation effects and the moths could be observed flying without noticeable effect of the X-ray exposure. Based on the highly repetitive movements, with an exact *n*-fold number of complete wingbeat cycles during the 1 s interval, a run of all radiographs would have generated the impression of freezing. The impression of movement arose from the complete series of wingbeat cycles running ahead by 1 s in relation to the X-ray frame rate. The small steps forward, repeated 300-fold, documented the movement, independent from the absolute number of cycles. Movie 1 shows consecutive frames 172 to 233 out of 300 and displays 10 visualised movements.

It is likely that the 300 images with 1 frame s^−1^ do not cover the minimum and maximum of the motion of the mesophragma and hence the total volume change of the air sacs. The radiographs were taken without simultaneous physiological recordings.

### Measurements of haemolymph flow

*Agrius convolvuli* (*N*=4) were used in the experiments. Measurements of haemolymph flow were performed with a thermistor glued onto the cuticle above the heart tube. The slight warming of the thermistor of a maximum of +0.35°C was sufficient to monitor the cooling effect of heart pulses below. Pulse direction within the heart tube was visualized by heat marking of the haemolymph flow. A glass fibre was used to project a He-Ne laser beam (+1.5°C; 12 V, 5 mW class 3, Conrad, Hirschau, Germany) onto the anterior abdomen. For measurements of heat-marked flow, thermistors (VECO Texas instruments) were glued onto the fifth abdominal segment above the heart tube and below the perineural sinus. Forward pulses – from the abdomen tip towards the thorax – led to cooling (forward) pulses at the fifth abdominal segment. Warming heart pulses measured at the posterior abdomen indicated warmed haemolymph flow from the anterior, which indicated reversed flow. In thermistor registrations below the PNS, enhanced flow during flight was documented by rising temperature, caused by flight muscle activity (method detailed in Wasserthal, 1980). The thermistors were also used for temperature measurement, but without the slight heating of the thermistor pearl.

In the figure text, specimen identification is given in parentheses by sex and numbered per year.

## RESULTS

### Increased *P*_O_2__ and mean tracheal pressure in the meso-scutellar air sacs after the transition from shivering to steady flight

The present study recorded air sac pressure and *P*_O_2__ by inserting two sensor tips simultaneously into the same artificial (meso-)scutellar hole of 1.5×1.5 mm (Fig. 2D) Remarkably, the *P*_O_2__ in the scutellar air sacs (SAs) during steady flight rose to nearly atmospheric partial pressure.

The moths spontaneously began to warm up by shivering under the simultaneous contraction of antagonistic flight muscles. At an ambient temperature of 20±1°C, the shivering lasted 2−3 min, while the *P*_O_2_ _declined to 9.35±3.23 kPa owing to O_2_ consumption (Fig. 2A,B). A steep rise occurred to nearly atmospheric *P*_O_2__ values under increasing pressure by approximately 30–50 Pa after switching from shivering to steady flight. This elevated O_2_ level persisted during flight. These effects are consistent in *M. sexta* (18.8*±*1.03 *P*_O_2__; *N*=6, *n*=125)_,_ *A. convolvuli (*19.4±0.2 *P*_O_2__; *N*=5, *n*=72) *A. atropos* (18.1±1.9 *P*_O_2__; *N*=6, *n*=34) and *X. morganii praedicta* (18.7 *P*_O_2__; *N*=1, *n*=18) (*n*=number of flight periods, length at least 3 min) (Table S1).

### Pressure and *P*_O_2__ measurements in the thoracic tracheal system and X-ray visualisation of volume changes

The tracheal volume and pressure conditions within the thorax responded to the alternative shortening of the dorsoventral and longitudinal flight muscles. The dorsal SAs and the fused air sacs of the metathorax and first abdominal segments (fused air sacs, FAS) are extensions of the tracheal trunks and must communicate.

Pressure measurements in the SAs during full flight showed higher amplitudes of pulses than during shivering, alternating from −150 to +170 Pa (Fig. 2A,B). Conspicuously, the average pressure level was 35–50 Pa higher than during shivering. This was correlated with the full-scale contraction of the dorsoventral muscles (Fig. 2C). The pressure pulses resulted from the augmented tension of the thoracic sclerites, which affects the power transmission from indirect flight muscles to the wings (Chapman, 2013; Gau et al., 2019). The lowered pressure down to −150 Pa is attributed to the buckling of the dorsal sclerites by the contracting DLM II, which induces the downstroke. Simultaneously, this must lead to the widening of the dorsal SAs. The negative pulses of −150 Pa during flight were correlated with −160 Pa at the inflow spiracle SP I (Wasserthal, 2001).

X-ray slow-motion movies during the tethered flight in *A. atropos* (*N*=3) documented the movement of the mesophragma and the fused metathoracic–abdominal air sacs. Shortening the dorsolongitudinal muscles during downstroke (Chapman, 2013) reduced the distance between the prothoracic and posterior attachments at the mesophragma. The phragma moved anteriorly, extending the fused air sacs. The impression of movement arose from the frequency of X-ray exposures lower than a multiple of the wingbeat frequency (see Materials and Methods). Movie 1 shows 10 thereby visualised protractions of the mesophragma, showing consecutive radiographs 172–233 out of 300, taken during 5 min with 1 frame s^−1^, exposures 5 ms. The moment of the downstroke is also visible by the shadow of the wing veins.

The flight muscles directly affected the closing and opening of the metathoracic spiracles. During every downstroke, the contracting dorsolongitudinal muscles protracted the lateral sclerites and closed the metathoracic spiracles in the metapleural cleft (Movie 2) (*A. atropos*, *N*=3) (Wasserthal, 2001). This allowed inflow only via the anterior spiracle.

Positive pressure pulses must be induced during the upstroke when the dorsolongitudinal muscles relax and the antagonistic dorsoventral muscles contract, compressing the thorax. This explains the rise in mean tracheal pressure by up to 50 Pa during full flight (Fig. 2). Because the lateral sclerites moved backwards during the upstroke, the metathoracic spiracles were now open, allowing outflow under elevated pressure. Their valve flaps reacted simultaneously but quicker than the slower closing and opening of the cleft (Movie 2).

During the flight, *P*_O_2__ fluctuations in the large thoracoabdominal air sacs differed from the continuously elevated O_2_ observed in the scutellar air sacs (Table S1). In *M. sexta*, *P*_O_2_ _in the thoracoabdominal air sacs during rest was about 9 kPa. During consecutive short flights of 15–20 s, a decline to 5–7 kPa was observed (Fig. 4A). The effect of the *P*_O_2__ decline was pronounced in the long run in *A. atropos* (Fig. 4B). During repeated flight bouts, ranging from 20 s to 17 min, every flight bout began with a short and transient rise of *P*_O_2__, followed by a sharp decline of decreasing *P*_O_2__. During 17 min of continuous flight, *P*_O_2__ lowered to a basic level of 5±1 kPa, including a post-flight period (Fig. 4B). The initial rise of *P*_O_2__ was interpreted as inflow of fresh air at the start of unidirectional airflow. The *P*_O_2__ decline after sustained flight indicated substitution by CO_2_, released from the haemolymph after O_2_ was partially depleted by flight muscle activity (Fig. 4A,B). The large thoraco-abdominal air sacs are near the metathoracic expiration spiracle and the CO_2_ output was measured only via these spiracles (Wasserthal, 2001).

### Anatomy of the thoraco-abdominal air sac

**Fig. 3.**
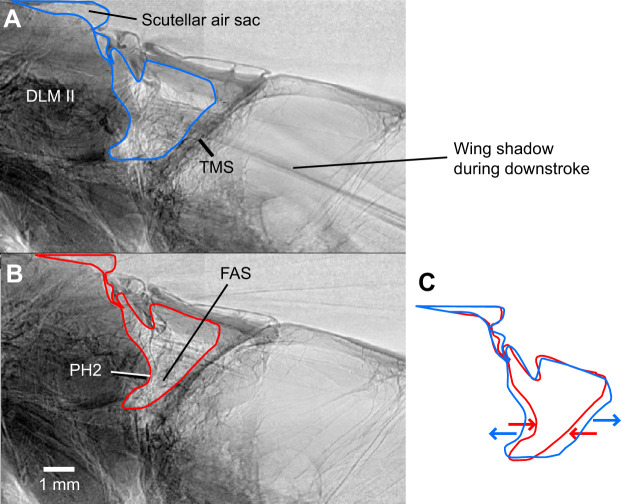
**Radiographs of the mesophragma shift inducing volume changes in the fused metathoracic–abdominal air sacs (FAS) by contraction and relaxation of the longitudinal flight muscles.** (A,B) Radiographs from the sequence of a tethered *Acherontia atropos* (male, 1/05) (Movie 1). Lateral view. (A) During downstroke of the wings, the contracting dorsolongitudinal muscles (DLM II) pull the mesophragma (PH2) in the anterior direction, widening the FAS under coordinated abdomen lifting (blue contours). (B) During upstroke of the wings, under contraction of the more lateral dorsoventral muscles, the relaxing DLM II allow backward shift of the PH2, reducing the space of the FAS (red contours). (C) Changes of bounds of the FAS, red and blue arrows indicating direction of volume change (redrawn from A and B). TMS, transverse muscle septum.

The whole-mount parasagittal cuts of the thorax showed continuity of the fused thoracoabdominal air sacs (*X. morganii N*=3, *A. atropos N*=2) (Fig. 1A,B) (chambre aerienne; Brocher, 1920). They are distended between the rear of the mesophragma and the transverse muscular septum (Hessel, 1969), occupying most of the space inside the metathoracic segment, including the first abdominal segment (Fig. 1B, dashed line). The corresponding view in the serial radiographs showed the inner right body side, visualising movements of the mesophragma (Fig. 3A,B). Owing to the continuity of these air sacs into the first abdominal segment within the joint region, abdominal movements must contribute to air sac extensions.

**Fig. 4.**
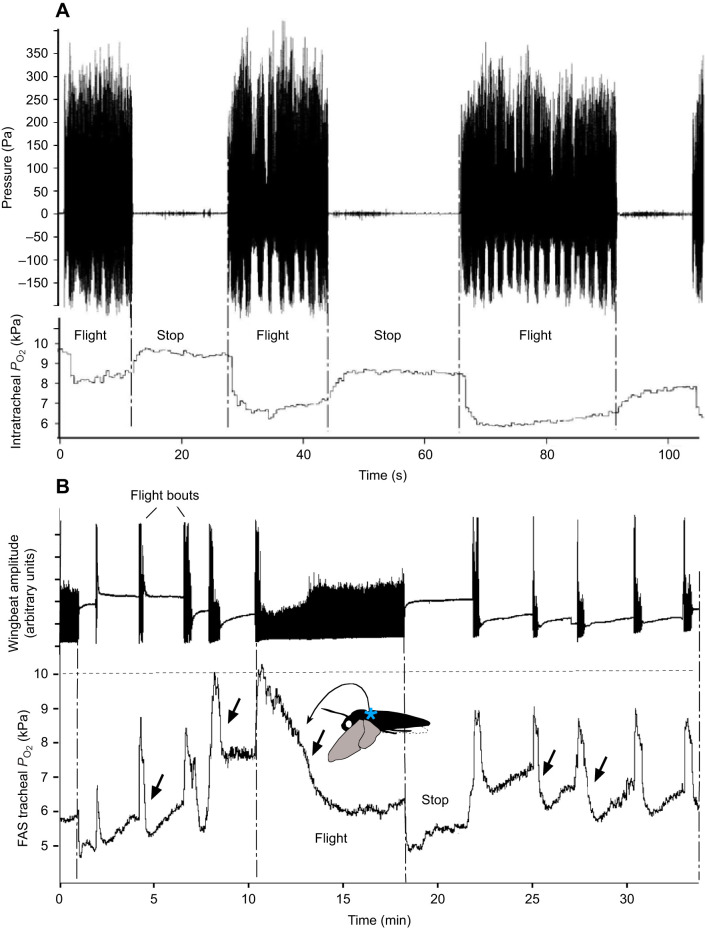
**In the fused metathoracic-abdominal air sacs (FAS), *P*_O_2__** **declines during flight.** (A) Measurements of pressure and *P*_O_2__ in the FAS in *Manduca sexta* (female, 6/04). Short flights of 10–24 s. Within the FAS, pressure pulses by wing muscle contractions moving the mesophragm (upper trace) are correlated with a *P*_O_2_ _decline from 9 to 6 kPa (lower line) (*n*=27). (B) The *P*_O_2__ in the FAS of *Acherontia atropos* (male, 1/18) over several minutes of flight and rest. Upper trace: photocell monitoring of the wingbeat. Lower trace: the *P*_O_2__ in the FAS at first rises with every flight bout, followed by a sharp decline (arrows). During continuing flights (A,B), *P*_O_2_ _drops to 6±1 kPa. Changes in the zero level of photocell monitoring were caused by slight positional changes in the moth. Blue asterisk, position of the FAS (*N*=3).

The fused air sacs are traversed by X-ray-dense strands (Fig. 1D–F). These correspond to strands with spiralised tracheal cuticles outside facing the air sac lumen and epithelia within the tubes (Brocher, 1920). The arrangement of these haemolymph channels suggests an increase in the tracheal surface for a more direct and efficient transition of the CO_2_, being buffered in the haemolymph, into the air sacs. This is important because the air sacs communicate with the metathoracic expiration spiracles. With X-ray CTs, we could not determine whether these channels were filled with haemolymph or contained only tissue. The brightest regions in Fig. 1F may also be a phase contrast effect. Comparable haemolymph channels were also described in the cephalic air sacs of ants and honeybees (Janet, 1911). Their structure needs further investigation.

### The thoracoabdominal air sacs are backed by the abdominal transverse muscular septum

The thoracoabdominal air sacs extended to the abdominal transverse muscle septum, formed by the tergosternal (dorsoventral) muscle fibres. The septum was between the first and second abdominal segments. The large fused air sacs were closely applied to the anterior surface of the transverse muscle septum (Fig. 1A–C) (Hessel, 1969). Its posterior surface is lined by the large air sacs of the second abdominal segment. This muscle septum must restrict the pressure pulses induced by the contracting flight muscles to the thoracoabdominal air sacs.

### Electrophysiological recording of abdomen flexing muscles and transverse muscle septum correlated with wingbeat cycle

The thoracoabdominal air sacs in the waist region must be directly affected by the flexing movements of the abdomen. With anatomic sections and electrophysiological records, we analysed a pair of dorsolongitudinal muscles of the first abdominal segment, which consist of two elements on each side. They connected the posterior phragma of the thoracoabdominal joint with the phragma of the first abdominal segment (Fig. 1). In the videos of tethered and free hovering moths, it is visible that the flexing movements were similar in every wingbeat cycle (*M. sexta N*=2, *M. stellatarum N*=6, *H. celerio*, *N*=20, *A*. *atropos N*=6) (Fig. 6; Movie 3). All species showed the same correlation, with abdomen down-bending during upstroke and lifting during downstroke.

**Fig. 5.**
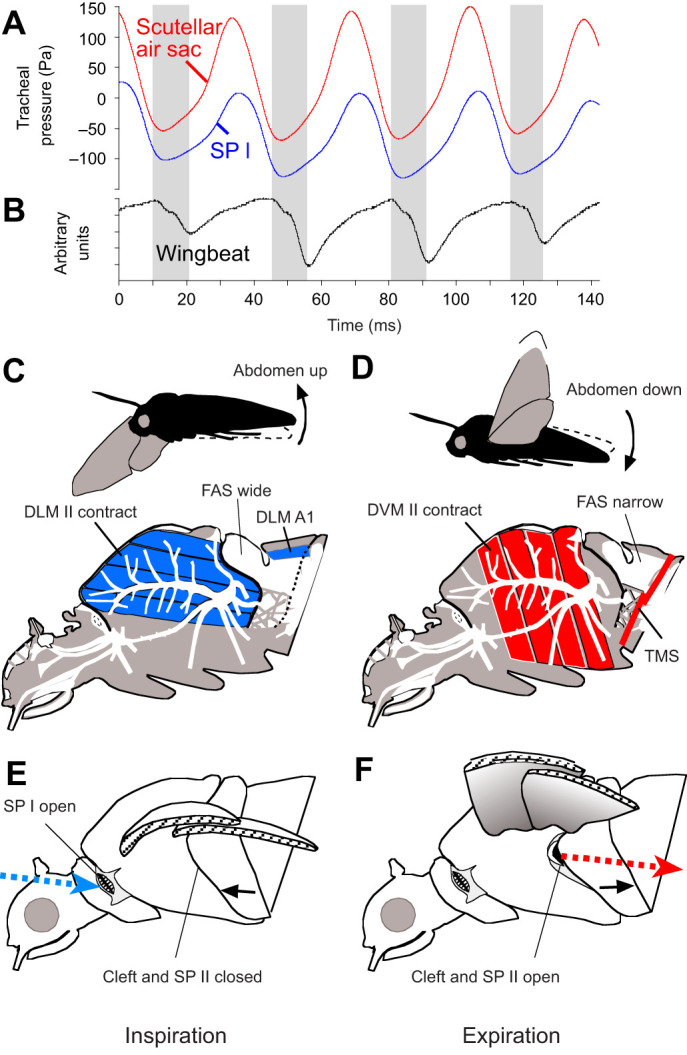
**Functioning of the flight apparatus and tracheal system, producing unidirectional respiratory airflow in flying hawkmoths.** (A) Tracheal pressure at spiracle I (SP I) during the wing stroke cycle is always lower than pressure in the scutellar air sac. (B) Wingbeat simultaneously recorded by photocell, grey bars indicate downstroke [A,B: *Manduca sexta* female, 1/01, monitored during the preceding study (Wasserthal, 2001)]. (C) Volume increase of the FAS during the downstroke, and (D) volume decrease during the upstroke. (E) Inspiration through the opened anterior spiracles during downstroke (SP I). (F) Expiration during upstroke through the second thoracic spiracles (SP II; Movie 2). C–F show a schematic view.

**Fig. 6.**
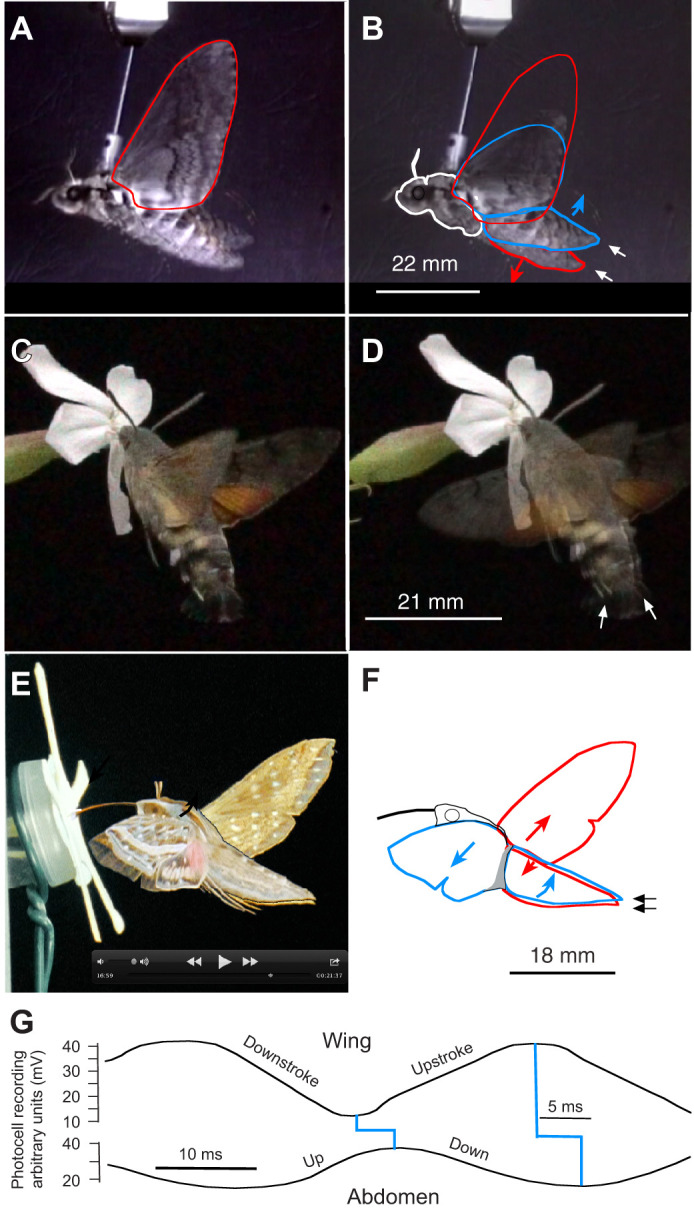
**Abdominal upward and downward flexing during the wingbeat cycle.** (A,B) Tethered flight of *Manduca sexta* male. Both pictures from same wingbeat cycle. (C,D) *Macroglossum stellatarum* female hovering before a *Saponaria* flower, with double contours at the posterior abdomen visible (arrows in D). (E,F) *Hippotion celerio* male hovering on the spot. Abdomen in up position (blue contours) and down position (red contours). Video frames show maximum and minimum deflection overlaid (B,D,E) (see also Movie 3 in *Acherontia atropos*). (G) Inverse positional changes of the wing and abdomen. Wing downstroke coincides with abdomen lift. Photocell and lateral reflex light trap recording of *H. celerio* (female, 3/90).

**Fig. 7.**
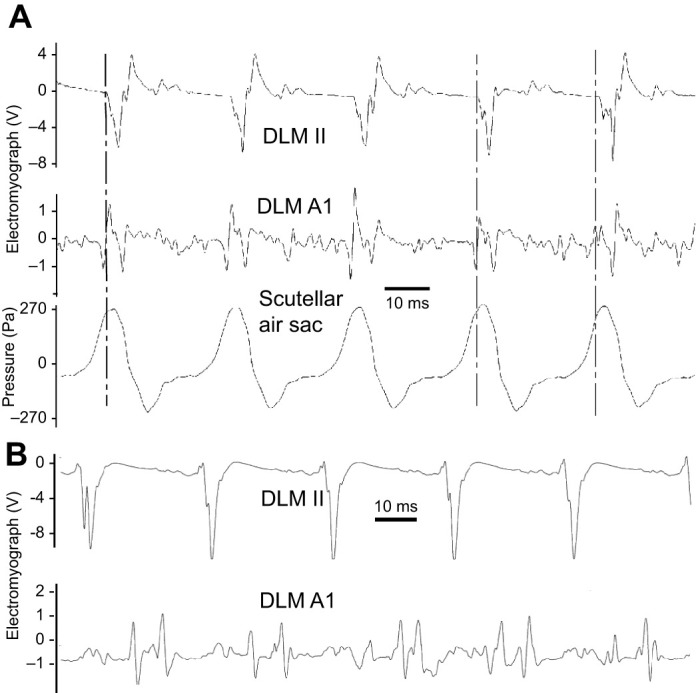
**During flight, the dorsolongitudinal flight muscles (DLM II) contract simultaneously with the dorsolongituinal muscles (DLM A1) in the first abdominal segment, inducing negative pressure pulses in the scutellar air sacs but alternate during shivering.** *Agrius convolvuli* (female, 2/03, *N*=3) electromyographs and pressure recordings. The DLM II induce the downstroke of the wings and move the mesophragma anteriorly, widening the air sac behind. The DLM A1 lifts the abdomen. (A) Action potentials of the wing depressor muscles DLM II and DLM A1 correlated with declining tracheal pressure pulses in the scutellar air sacs. (B) During warm-up shivering, contractions of DLM II and DLM A1 alternate. This is correlated with a lower level of scutellar pressure (see Fig. 2).

**Fig. 8.**
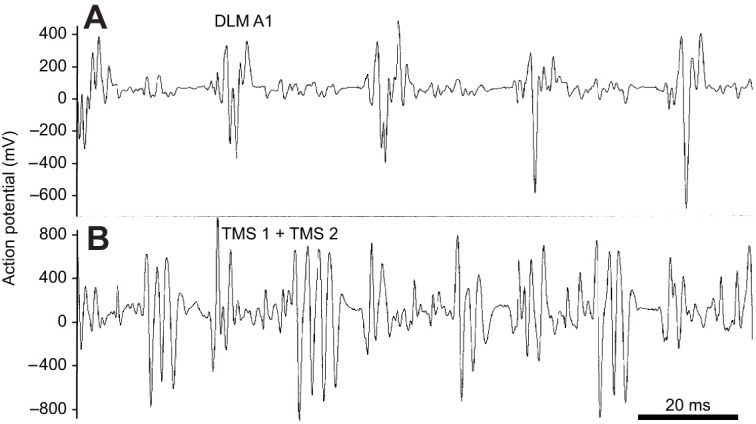
**The transverse muscle septum in the anterior abdomen is active during the upstroke and downstroke of the wings.** Electromyographs of the (A) abdominal flexing muscles (DLM A1) and (B) transverse muscle septum (TMS 1 and TMS 2) in *Agrius convolvuli* (male, 2/03, *N*=3).

Simultaneous electrophysiological recordings of these abdomen lifting muscles – considered homologous with the longitudinal flight muscles – were performed in *A. convolvuli* (*N*=3). Their simultaneous activity confirmed the direct coupling of abdominal lifting by these muscles and downstroke (Fig. 7A). During shivering, these muscles contracted alternately (Fig. 7B). In contrast, electrophysiological recordings of the muscles in the abdominal transverse septum documented their activity during upstroke and downstroke (*A. convolvuli N*=3) (Fig. 8). These smaller muscles may correspond to the dv1 and dv2 described by Eaton (1988).

### Thermistor measurements of haemolymph flow in the dorsal heart tube and through the perineural sinus during flight

**Fig. 9.**
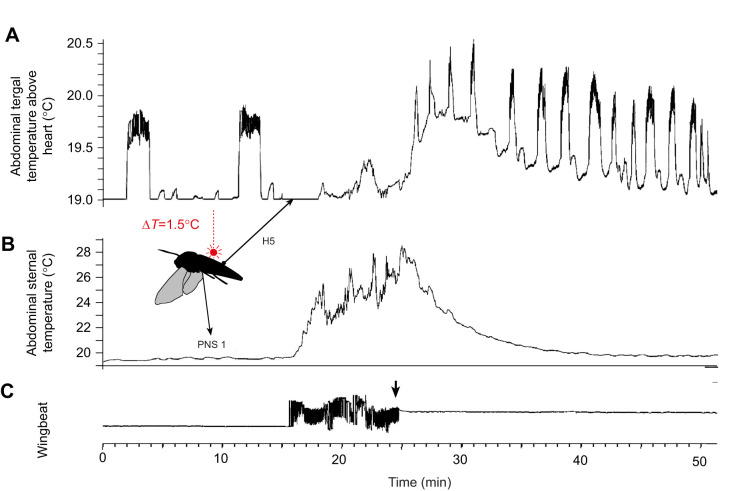
**The direction of haemolymph transport in the dorsal heart tube changes from periodic heartbeat reversals during rest to continuous forward pulses during flight, with enhanced flow in the perineural sinus (PNS), visualised by thermistor recording.** *Hippotion celerio* (male, 1/00, *N*=4). (A) Abdominal tergal temperature above the heart (H5, fifth segment). (B) Abdominal sternal temperature. (C) Photocell registration of the wingbeat. At rest, periodic backward haemolymph transport is documented by warming pulses under heat marking above the heart tube by +1.5°C at the anterior abdomen and a measuring thermistor posterior on the fifth abdominal segment. During 9 min of flight, the thermistor recording shows a lower temperature with weak and irregular pulses. This indicates forward pulses with unheated haemolymph from behind without intermittent backward beating. Simultaneously, the enhanced ventral haemolymph flow into the abdomen, warmed by flight muscle activity, is indicated by the rising temperature in the PNS.

Periodic heartbeat reversals are characteristic of resting Lepidoptera (Wasserthal, 1980, 1982). Heartbeats were recorded in *H. celerio* (*N*=4) by applying a thermistor to the cuticle above the abdominal heart tube and ventrally below the perineural sinus under dorsal warming of the anterior abdomen by a laser beam of +1.5°C (Fig. 9).

At rest, with anterior abdominal heat marking, thermistor recordings on the fifth abdominal segment showed a periodic sharp rise in temperature above the dorsal heart tube, indicating intermittent reversed flow. During the flight, these reversals stopped. The thermistor recordings showed a lower temperature level. Single pulses were not resolved because the pulses did not pass a heated area before passing the measuring thermistor. This indicated that the heartbeats during the flight were continuously forward. Simultaneously, the enhanced continuous flow backwards through the perineural sinus was documented by the rising temperature at the ventral abdomen below, indicating a haemolymph flow warmed up by flight muscle activity (Fig. 9). The temperature on the thorax rose to 35 to 37°C during full flight. The value was variable and dependent on the moment of warm up shivering, when the moths start to fly (see also Heinrich and Bartholomew, 1971)

## DISCUSSION

### Sphingids have developed unique functional traits for flight muscle-driven ventilatory airflow

The high-performance flight and energy demand in sphingids leads to the quest to understand the functional adaptations for unidirectional ventilation.

Two distinct types of tracheal ventilation of flight muscles have been described in insects. Abdominal pumping is a well-known mechanism performed with low-frequency pulses, e.g. in the hymenopteran *Vespa* (Weis-Fogh, 1964a). The other type is thoracic pumping with a high wingbeat frequency. Thoracic pumping appears to be the primary mechanism for ventilation of the thorax during flight in grasshoppers, dragonflies, moths and beetles (Weis-Fogh, 1964a). The movements of the nota and pleura, which accompany the wing movements, are assumed to ventilate the thoracic air sacs as a kind of autoventilation in and out of all spiracles (Miller, 1960) (Weis-Fogh, 1964b). Wingbeat frequencies of about 27.3–73 Hz in hawkmoths have been described by Weis-Fogh (1973). The shortening and relaxing of the thoracic flight muscles must lead to muscular pumping (Weis-Fogh (1964b). Weis-Fogh assumed that only an intricate system of unidirectional valves in the primary tracheae could ensure an adequate blood supply and air flow in and out, stating that such valves were not known (Weis-Fogh, 1964b).

In contrast, an alternative model with unidirectional flow has been described in the blowfly *Calliphora* with inward flow only at the anterior thoracic spiracles and outflow through the metathoracic spiracle: intact spiracles were tubed and connected to separate chambers, measuring CO_2_ output by flow-through respirometry. CO_2_ outflow during the flight was only through the posterior spiracles (Wasserthal, 2015).

This paper is based on documenting unidirectional tracheal ventilation in flying sphingids in a preceding publication: the moths were enclosed in a vessel, using flow through CO_2_ respirometry. The tubes sealed onto the anterior spiracles were connected to a separate chamber. CO_2_ output was registered only in the chamber with the posterior thoracic spiracles and abdomen (Wasserthal, 2001) (for details, see Introduction).

Anatomical adaptations must exist to direct ventilatory flow. In sphingids, an intricate valve mechanism has been described, as postulated by Weis-Fogh (1964b), as the precondition for unidirectional flow (Wasserthal, 2001). The valve function is performed by closing the metathoracic spiracles in the intersegmental cleft when the DLM II protracts the lateral sclerites (Movie 2), allowing inspiration only via the anterior spiracle. This leads to high *P*_O_2__ values in the SAs below the buckling dorsal sclerites up to 18.7±1.67 kPa (Fig. 2; Table S1). The release of CO_2_ loaden air was possible only during wing upstroke through the metathoracic spiracles. The contracting dorsoventral flight muscles – under relaxing of the dorsolongitudinal flight muscles – now shift the lateral sclerites backwards, exposing the metathoracic spiracles, which open in the intersegmental cleft (Movie 2). In contrast, Komai (1998, 2001) stated that *A. convolvuli* has no large air sacs and does not use unidirectional ventilation flow. Komai describes augmented respiration during flight with *P*_O_2__ values between 6.27 and 0.10 kPa in measurements within thoracic flight muscles, which vary more depending on the positional differences of the sensor tip, whether it was located more or less profoundly intramuscular or intercellular. The intramuscular *P*_O_2__ values were lower than those inside the air sacs found in the present study.

The unidirectional airflow works only under full-flight conditions. During pre-flight warm-up (shivering), starting at the environmental temperature of 20±1°C, *P*_O_2__ decreased to half the value of steady flight with lower pressure amplitude in the scutellar air sacs (Fig. 2). This showed that during shivering when some antagonistic flight muscles contract simultaneously (Kammer, 1968) and the abdominal flexing muscles are out of phase (Fig. 7), O_2_ consumption is not compensated by ventilatory flow (Fig. 2A,B)

### Fused thoracoabdominal air sacs form a tracheal bellow, supporting unidirectional airflow

In this study, the bellow-like air sacs in the waist provide central compressible and extensible volumes, facilitating flow-through ventilation in Sphingidae. The functional role of these metathoracic–abdominal air sacs was revealed by their widening under protraction of the cuticular elastic mesophragma by the longitudinal flight muscles. The role of mesophragma in tracheal ventilation has already been discussed for *Bombus* (Miller, 1981). The anatomical configuration alone suggests the widening of these air sacs under contraction of the dorsolongitudinal flight muscles (Fig. 1) and was documented in *A. atropos* based on 300 X-ray frames taken over 5 min (Fig. 3; Movie 1). This pumping by flight muscle contractions appears to directly correlate the ventilatory flow with wingbeat frequency and amplitude, which requires further quantitative evaluation.

The bending movements of the abdomen are an integral part of the wingbeat cycle and are not used only for steering (Dyhr et al., 2013). This is obvious under free-flight conditions, allowing positional changes in the abdomen (Fig. 6C–G). The tracheal space of the fused air sacs extending into the anterior abdomen must also ease the mobility of the flight muscles and the joint region.

### Coordination of the flight muscles, abdominal flexing muscles and transverse muscle septum affects the suction generation for ventilatory flow

The dorsolongitudinal and dorsoventral flight muscles generate wing depression and wing elevation, respectively (Wendler, 2003; Deora et al., 2017). The abdomen lifting muscles and the muscles of the transverse septum have not yet been investigated, although general involvement in abdomen flexing is assumed. Real moths control the relative abdominal angle through the actions of muscles at the thoracoabdominal joint (Dyhr et al., 2013). Additional muscles are described in the first abdominal segment and around the thoracoabdominal joint (Eaton, 1988), but their function has not been analysed.

The smaller dorsolongitudinal muscles in the first abdominal segment, described here as abdomen lifting muscles, are regarded as homologous with the thoracic longitudinal flight muscles, deduced from their similar arrangement and simultaneous contraction with the longitudinal flight muscles during steady flight. Their activity correlates with the pressure decline in the SAs following a preceding pressure rise (Fig. 7A). This means the preceding positive pressure pulses – attributed to contractions of the dorsoventral muscles – supplied the suction flow towards the fused air sacs. Thus, the suction pulses by the contracting dorsolongitudinal muscles intertwine with contractions of the dorsoventral muscles, which compress the thorax during the upstroke. Lifting of the abdomen must support the sucking effect in the tracheal system by widening the air sacs in the waist. It must also facilitate haemolymph flow through the perineural sinus.

The abdominal transverse muscular septum (Hessel, 1969) must have a functional impact in thoracic ventilation. The septum with the two pairs of muscles can be understood as an antagonist to the abdomen lifting muscle. The electromyogram with activity synchronous to dorsolongitudinal flight muscles and additional intermittent bursts (Fig. 8) showed that the muscle fibres within the septum were activated during the upstroke and downstroke of the wings. This suggests controlled stiffness, which prevents the transmission of pressure fluctuations within the thoracoabdominal air sacs towards the large abdominal air sacs. The septum thus aids in pressure compartmentalisation (Pendar et al., 2019; Wasserthal, 1996), supporting the ventilation outflow only via the metathoracic spiracle (Fig. 5).

The pressure gradient driving the ventilation flow was evaluated in split-chamber flow-through experiments (Wasserthal, 2001). A pressure increase was applied stepwise within the posterior CO_2_ outflow chamber to reverse ventilatory flow. In *M. sexta*, the reversal with CO_2_ emission from the anterior thoracic spiracle began under a pressure rise in the posterior chamber by +25 Pa. An imposed counter pressure of +50 Pa further increased the CO_2_ outflow anteriorly. However, remarkably, this was during flight pauses, showing that the flight motor had worked against the diffusive outflow from the anterior spiracles (Wasserthal, 2001). Thus, the tracheal meso-scutellar pressure levels, rising to mean pressure levels of 35–50 Pa during full flight in *M. sexta* and *A. convolvuli* (Fig. 2), represent the overall pressure rise within the tracheal system for efficient flow-through ventilation.

The ventilatory flow did not work during pre-flight warm-up shivering, with simultaneous but reduced contractions of dorsolongitudinal and dorsoventral flight muscles (Fig. 2) (Kammer, 1968), i.e. when the abdominal lifting muscles did not contract alternatingly with the dorsolongitudinal flight muscles (Fig. 7B). Thus, they did not contribute to expanding the thoracoabdominal air sacs.

### Switching from periodic heartbeat reversals to continuous circulation supports the removal of CO_2_ via air sacs

In different insect orders, periodic heartbeat reversals are characteristic of haemolymph circulation (Wasserthal, 1980, 1982, 1996, 1999). In *Calliphora*, which lacks an abdominal perineural sinus (Richards, 1963), heartbeat reversals continue during flight with augmented frequency (Wasserthal, 2015). In contrast, in Sphingidae, during flight, the haemolymph exchange between the thorax and abdomen switched from periodic heartbeat reversals to continuous circulation through the forward beating heart dorsally, with increased flow backward ventrally through the perineural sinus (Fig. 9). These results confirm earlier reports. In *Manduca*, enhanced forward pumping of the heart, combined with a more vigorous flow through the perineural sinus, was described as a mechanism for thoracic temperature stabilisation during flight (Heinrich, 1970). Heinrich (1970) described a weak and irregular heartbeat between 20 and 30°C after flight activity. Regular heartbeat reversals during rest were investigated in *Manduca* by Dulcis et al. (2001).

The ventral haemolymph flow through the perineural sinus is suggested to be eased by abdominal lifting during downstroke. A more vigorous haemolymph circulation must support the CO_2_ output via the fused thoracoabdominal air sacs and abdominal air sacs during flight. It may also serve for distributing carbohydrate and lipid substrates activated during flight (Liebrich and Gäde, 1995; Surholt et al., 1991; O'Brian and Suarez, 2001).

### Conclusions

In hawkmoths during flight, thoracic unidirectional ventilatory inflow via the first thoracic spiracles depends on thoracic volume changes by alternating contractions of the dorsoventral and longitudinal flight muscles. The contracting longitudinal muscles during downstroke induce the buckling of the thoracic box and move the lateral sclerites anteriorly, enclosing the metathoracic spiracles in the intersegmental cleft (Movie 2) (Wasserthal, 2001). Thus, ventilatory inflow is possible only via the anterior spiracle. This leads to a rising *P*_O_2__, measured in the SAs.

The internal movement of the vertical cuticular mesophragma supports the unidirectional flow mechanism. The posterior attachment structure of the longitudinal flight muscles under contraction moves anteriorly, widening the air sacs behind. Because the contracting longitudinal flight muscle induces the closing of metathoracic spiracles, the air stream sucks from the anterior tracheal system. During the upstroke, the longitudinal flight muscles relax, and the mesophragma shifts backwards, lowering the volume of the air sacs behind. Simultaneously – under pressure imposed by the dorsoventral flight muscles – the pleural clefts are opened and the metathoracic spiracles near the thoracoabdominal air sacs allow the outflow of CO_2_ (Wasserthal, 2001) (Fig. 4). The path for CO_2_ removal is separated from the intratracheal flow of fresh air by buffering in the haemolymph (Chapman, 2013; Chown and Nicolson, 2009). The accumulating CO_2_ must pass from the haemolymph into the gas phase, diffusing through the tracheal walls near the spiracles (Harrison et al., 1995). The fused thoracoabdominal air sacs must facilitate conveying CO_2_ towards metathoracic outflow spiracles.

Serial X-ray synchrotron frames of flying *A. atropos* provide evidence for the functioning of the bellow-like pumping mechanism (Movie 1; Fig. 3). The thoracoabdominal air sacs function as the central device to use the power of the flight muscles to drive the flow-through ventilation in sphingids. This ventilation mode facilitates raising energy expenditure to a 148-fold O_2_ consumption from rest to full flight (Bartholomew and Casey, 1978). In *Calliphora*, another flight ventilation mechanism exists, with high frequency (145 Hz) but low volume changes (about 1%) of the thorax, generating directed flow with the support of an intratracheal fold acting as a backpressure valve, as documented by X-ray tomography (Wasserthal, 2015; Wasserthal et al., 2018).

A comparison with hummingbirds reveals the efficiency of unidirectional ventilation in insects. The metabolic rate of 237 mW g^−1^ body mass in *M. sexta* (Casey, 1976) equals the energy expenditure of the hummingbird *A. fimbriata* 232 mW g^−1^ (Berger and Hart, 1972). When comparing the ventilation volume of 30 ml g^−1^ min^−1^ in hummingbirds (Berger and Hart, 1972) with 35.08 ml g^−1^ min^−1^ in male *Calliphora* and 26 ml g^−1^ min^−1^ in female *Calliphora*, it was shown to be of the same scale (Wasserthal et al., 2018). Birds need extra ventilatory muscles, which work under the load of flight muscles (Funk et al., 1992; Tucker, 1972). As a fundamental difference, the contracting flight muscles in insects directly affect the tracheal ventilation. The calculation of the flow-through volume for *Calliphora* was made in a complementing X-ray tomographic analysis based on wingbeat frequency and amount of thorax shortening per wingbeat (Wasserthal, 2015; Wasserthal et al., 2018).

An X-ray tomographic reconstruction of the thoracic tracheal system will be needed in further studies in sphingid moths to visualise the tracheal pathway and calculate volumetric ventilatory flow. The maximal volumetric flow must be analysed under forced flight, e.g. by fanning or moving patterns.

Recently, Harrison et al. (2023) have provided an overview about air sacs in insects as an adaptive trait in the insect respiratory system. Generally, insect tracheal ventilation relies on ventilatory muscles contracting the exoskeleton. The present paper describes a new mechanism for the ventilation of air sacs: the contracting dorsolongitudinal flight muscles attached to the mesophragma directly exert pumping movements to ventilate the airsacs behind, working like a piston. A comparable unidirectional ventilation mechanism driven by flight muscles attached to a mesophragma may be discovered in other insects. Large air sacs on the rear of the phragma suggest such a mechanism. Also, filter structures at anterior spiracles, contrasting with missing or weak filters at posterior spiracles (Wasserthal, 2001; Wasserthal and Fröhlich, 2017), may indicate inflow versus outflow and suggest unidirectional flow.

## Supplementary Material

10.1242/jexbio.245949_sup1 Supplementary information
