# Supplementary material for: Functioning of unidirectional ventilation in flying hawkmoths evaluated by pressure and oxygen measurements and X-ray video and tomography
Source: J Exp Biol. 2024 Sep 17;227(18):jeb245949. doi: 10.1242/jeb.245949 (PMC11418177; doi:10.1242/jeb.245949)
Supplement: Supplementary information [file jexbio-227-245949-s1.pdf]

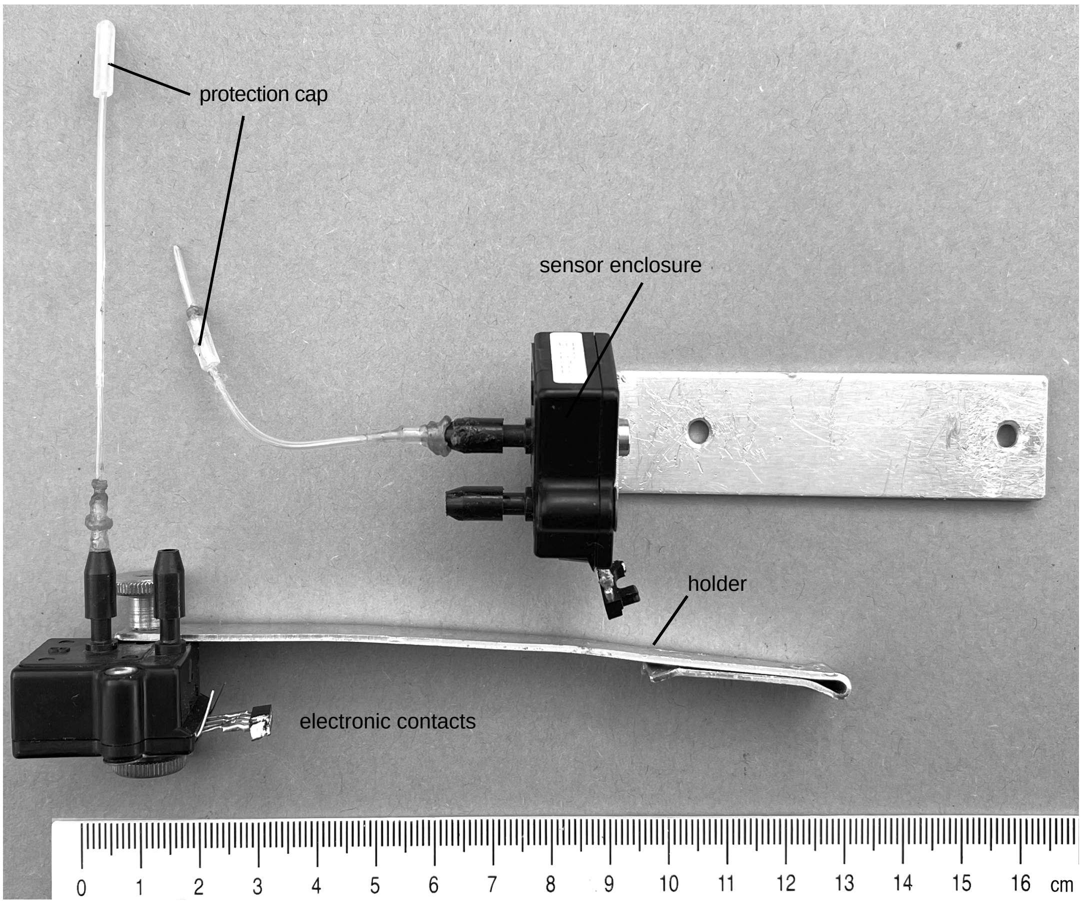

Fig. S1. Pressure sensors SCXL 004 DN, Honeywell, equipped with microcatheter PVC tubes, diameter 1.2 mm, internal diameter 0.8 mm, used for insertion into the adapter cone (Fig. 2D).

Table S1. The partial pressure of oxygen in the scutellar air sacs of tethered hawkmoths during shivering and voluntary steady state flight of at least 3 min, n = number of flight periods. (♂) male, (♀) female.

Species	No	recorded Flight periods	PO_2 (kPa) Shivering	PO_2 (kPa) Flight
Acherontia atropos	♂1/05	5	11.0	18.5
Acherontia atropos	♀1/05	5	7.5	15.0
Acherontia atropos	♀1/07	7	0.7	19.0
Acherontia atropos	♂2/05	5	14.0	19.7
Acherontia atropos	♂3/05	5	14.5	16.5
Acherontia atropos	♂1/18	7	0.5	20.0
Total		34	9.0 ± 5.2	18.1 ± 1.9
Agrius convolvuli	♂2/03	33	8.0	16.0
Agrius convolvuli	♀5/03	7	11.0	20.5
Agrius convolvuli	♀3/03	18	10.0	19.5
Agrius convolvuli	♂4/03	11	9.5	20.0
Agrius convolvuli	♀6/04	3	6.5	21.0
Total		72	9.0 ± 1.7	19.4 ± 0.2
Manduca sexta	♂2/04	4	9.0	20.0
Manduca sexta	♂4/04	11	10.0	18.0
Manduca sexta	♂5/04	15	8.0	17.5
Manduca sexta	♀6/04	37	9.5	18.5
Manduca sexta	♀3/04	32	10.0	20.0
Manduca sexta	♂3/04	26	13.0	19.0
Total		125	9.91 ± 1.68	18.8 ± 1.03
Xanthopan	♀1/02	18	9.35	18.7

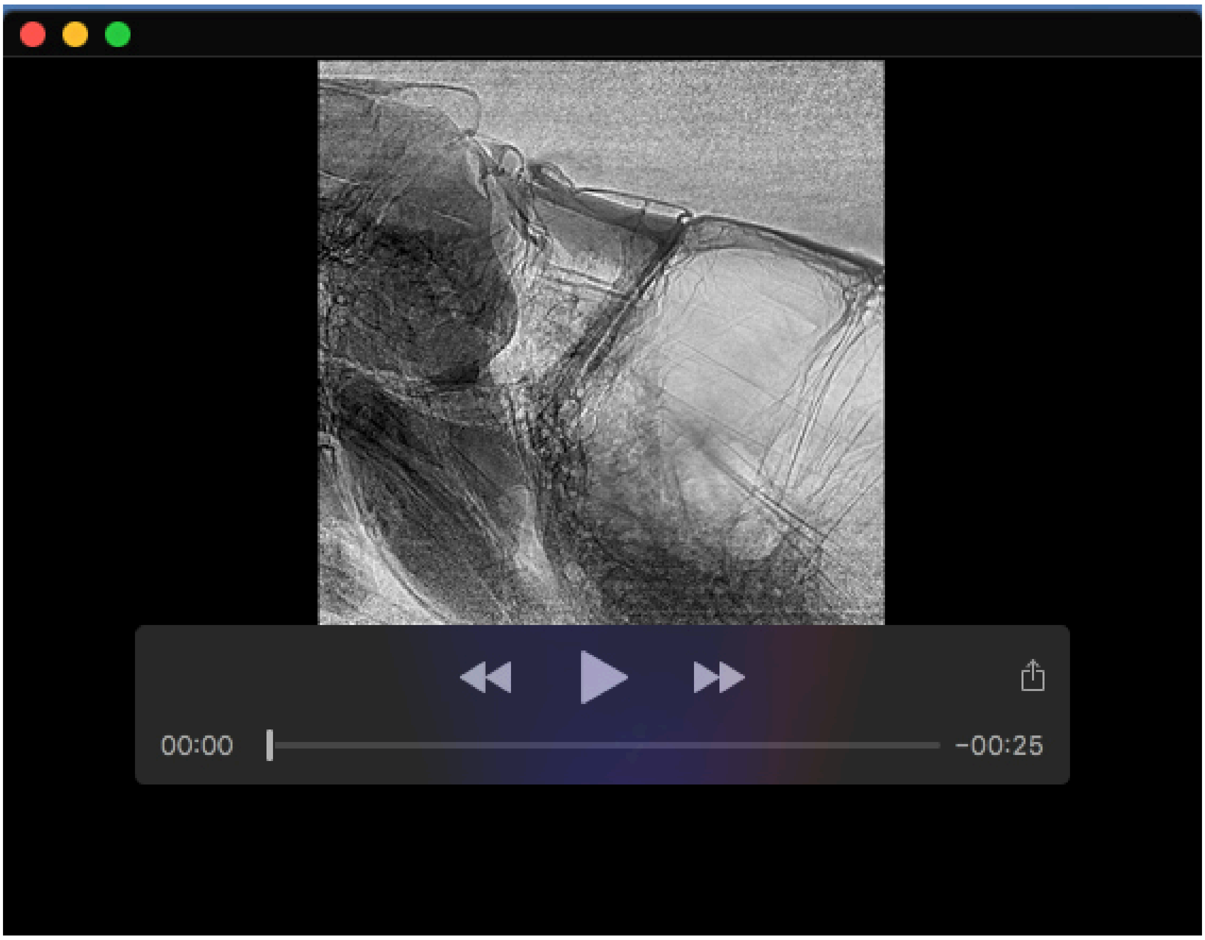

Movie 1. ***Acherontia atropos:*** **X-ray video of metathoracic-abdominal air sacs.** Protractions of the mesophragma by DLM flight muscles generating volume changes of air sacs, visualised by consecutive radiographs 172 – 233 out of 300 exposures during 5 min (see Materials and Methods), showing 10 protractions. 18 mb.

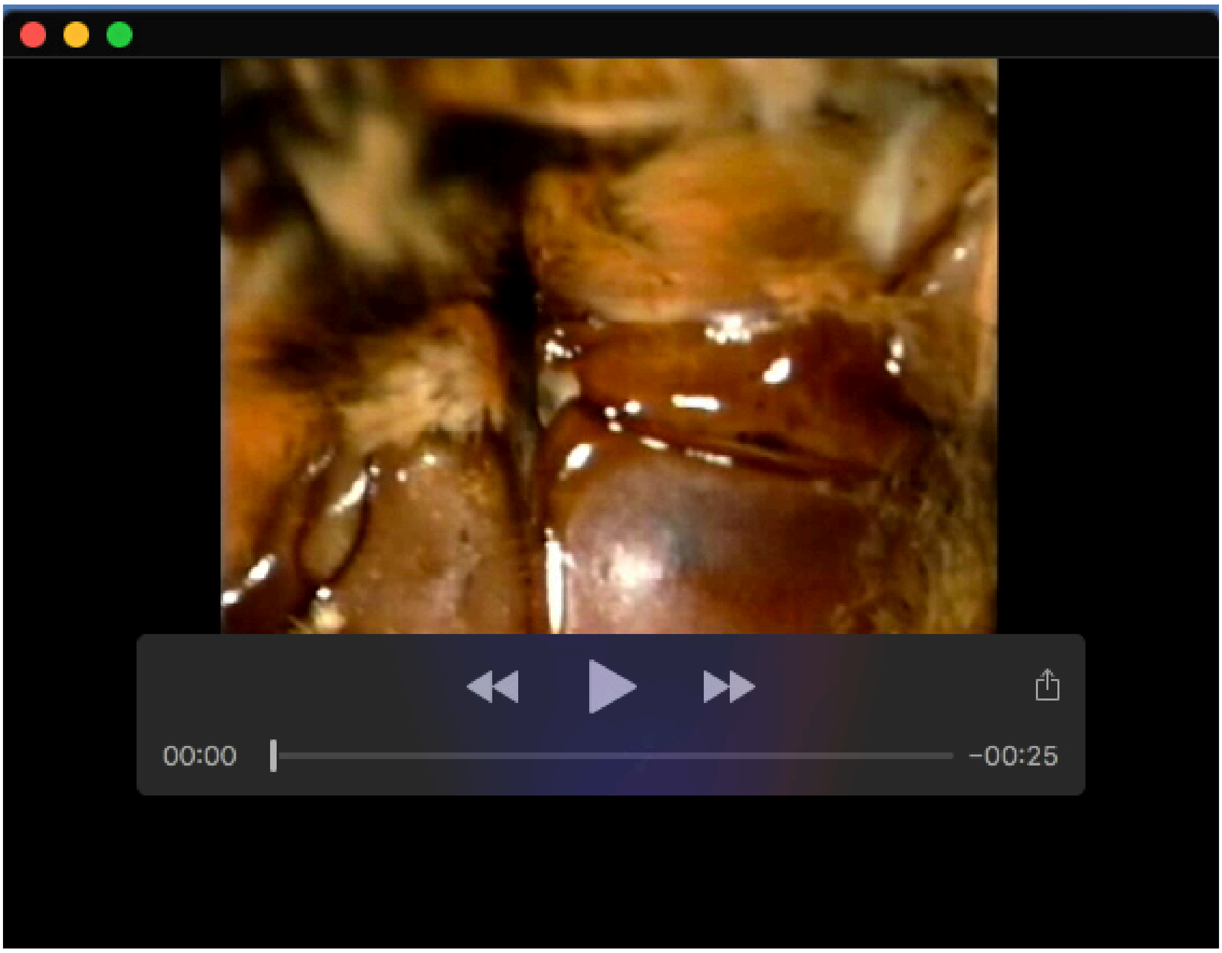

Movie 2. *Acherontia atropos*. During downstroke the metathoracic spiracle under contraction of the dorsolongitudinal muscles is enclosed in the intersegmental cleft. Inversely during upstroke under contraction of the dorsoventral muscles the lateral sclerites are moved backwards and the spiracle opens. Sony Alpha 9, slow motion option. The recording frame rate of 30 fps was slightly lower than the wing beat frequency of the moth 30. 24/s almost freezing the movements, generating a slow-motion effect (see Materials and Methods).

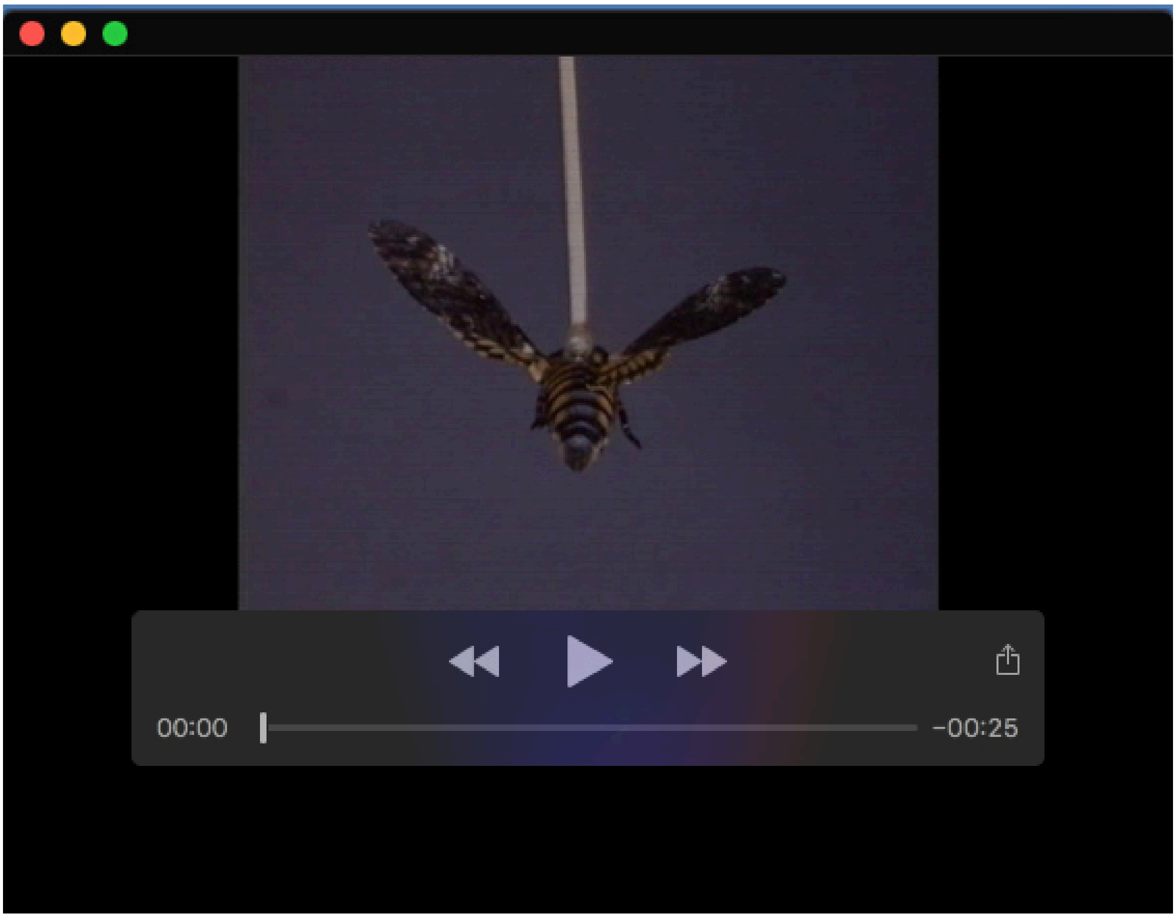

Movie 3. ***Acherontia atropos,*** **abdomen lifting during the downstroke, which affects ventilation of the thoracoabdominal air sacs.** View from behind**,** tethered spontaneous flight under dimmed light, 11 wingbeat cycles. Sony alpha 9. Slow motion option 25 frames /s, frame rate adjusted to the wing beat frequency to almost freeze the movements, generating a slow motion effect.
